# Retraction: Critical review of the evidence for Vojta Therapy: a systematic review and meta-analysis

**DOI:** 10.3389/fneur.2026.1971337

**Published:** 2026-08-24

**Authors:** Frontiers Editorial Office

The journal retracts the article cited above.

Following publication, concerns were raised regarding the scientific validity of the article. An investigation was conducted in accordance with Frontiers' policies.

The authors failed to provide a satisfactory explanation and as a result, the conclusions of the article have been deemed unreliable and the article has been retracted.

This retraction was approved by the Chief Editors of *Front. Neurol. – Neurorehabilitation* and the Chief Executive Editor of Frontiers. The authors do not agree to this retraction.

Frontiers would like to thank Alfredo Lerín, Selma Peláez, Lourdes Macías, Mónica Alonso, Marcos Moreno Verdú, Mijna Hadders-Algra and the other concerned readers who contacted us regarding the published article.

